# Application of Design of Experiment and Simulation Methods to Liquid Chromatography Analysis of Topical HIV Microbicides Stampidine and HI443

**DOI:** 10.4172/2155-9872.1000180

**Published:** 2014-01-24

**Authors:** Vivek Agrahari, Jianing Meng, Tao Zhang, Bi-Botti C Youan

**Affiliations:** Laboratory of Future Nanomedicines and Theoretical Chronopharmaceutics, Division of Pharmaceutical Sciences, School of Pharmacy, University of Missouri-Kansas City, USA

**Keywords:** Stampidine, HI443, Experimental design, Monte Carlo simulation, Defect rate

## Abstract

This study intended to determine if experimental design and Monte Carlo simulation methods can be utilized to optimize the liquid chromatography (LC) analysis of active molecules. The method was applied for the simultaneous analysis of two topical microbicides, stampidine (STP) and HI443 in bulk and nanoformulations. The Plackett-Burman design was used for screening; whereas, Box-Behnken design was used to evaluate the main and interaction effects of the selected factors on the responses, namely peak area of STP (Y_1_), HI443 (Y_2_), tailing of STP (Y_3_), and HI443 (Y_4_). The Monte Carlo simulation was applied to get the minimum defect rate (DR) of the process. The optimized LC conditions were found to be X_1_; flow rate: 0.6 mL/min, X_2_; injection volume: 18 μL, and X_3_; initial gradient acetonitrile ratio: 92% v/v with a minimal DR of 0.077%. The optimized method was applied to determine the percent encapsulation efficiency (%EE) and *in vitro* release profile of STP and HI443 from solid lipid nanoparticles (SLNs). The %EE of STP and HI443 in SLNs was found to be 30.56 ± 9.44 and 94.80 ± 21.90% w/w, respectively, (n=3). It was observed that the release kinetics of STP followed the first order, whereas, HI443 followed the Peppas kinetic model in SLNs. The LC method was also applied for the estimation of molar extinction coefficients (*ε_270_*) of both drugs for the first time. These values were estimated to be 7,569.03 ± 217.96 and 17,823.67 ± 88.12 L/mol/cm for STP and HI443, respectively, (n=3). The results suggest that experimental design and Monte Carlo simulation can be effectively used to reduce the DR of a process and to optimize the chromatographic conditions for the analysis of bio-active agents as applied in this study.

## Introduction

The design of experiment is now getting much attention in analytical method development [1–4]. The US Food and Drug Administration (FDA) and the International Conference on Harmonization (ICH) guidelines (Q8, Q9 and Q10) recommend the use of the design of experiment approach in analytical methods development [5–8]. Traditional approaches for method development involve the time consuming process of varying one factor at a time and examining its effect, which requires a large number of experimental runs. The defect rate (DR) of the process can be analyzed using simulation methods in combination with the experimental design approach. The DR analysis determines how many errors in the process occur on average with respect to variation in the factors in the experimental design. Thus, this study intended to determine that experimental design and simulation methods in combination can be utilized to optimize the assay conditions as applied to the analysis of two topical microbicides stampidine and HI443.

**Figure 1:**
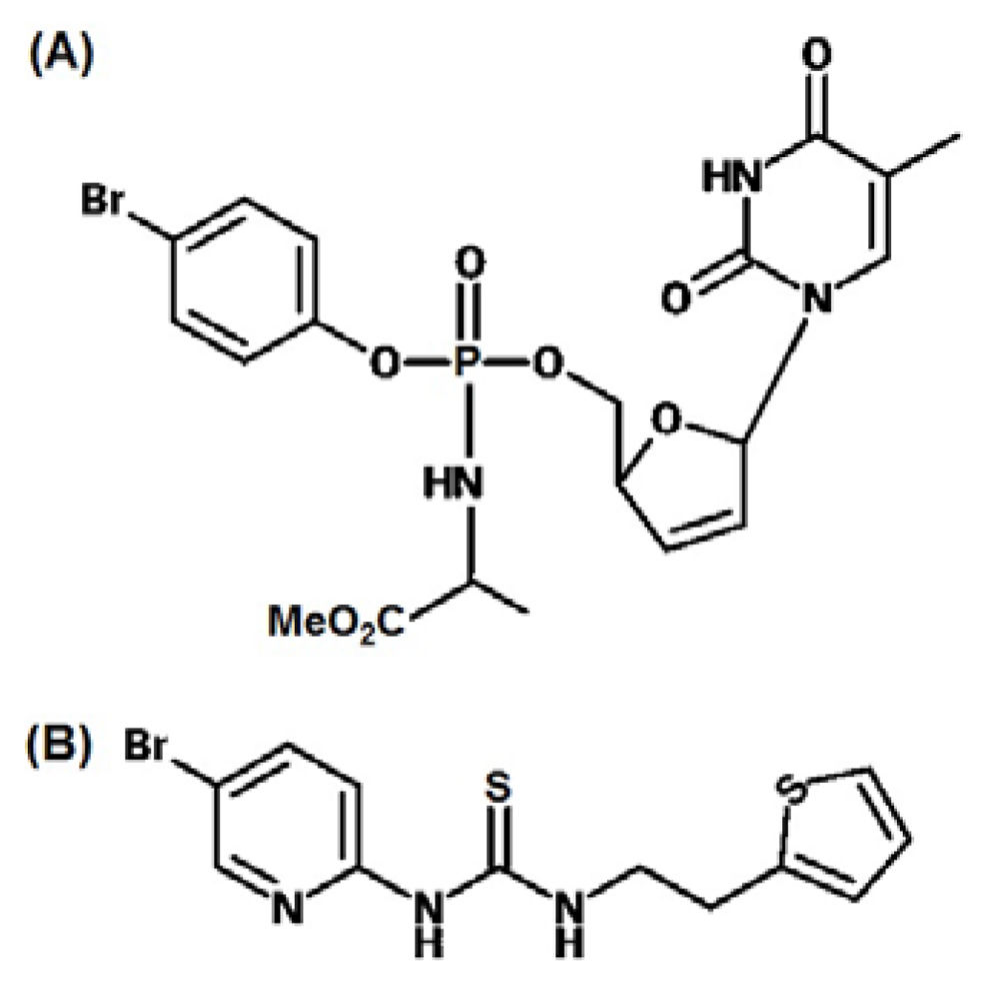
Chemical structures of (A) Stampidine: STP (B) HI443.

Stampidine (STP) is a nucleoside reverse-transcriptase inhibitors (NRTIs) based microbicide (Figure 1A) and a phosphoramidate derivative of stavudine [9]. It exhibits broad-spectrum anti-HIV activity in nanomolar to submolar concentration and has the potential to abrogate all the steps in the life cycle of HIV virus [9,10]. Also, due to the lipophilic nature of STP (log P: 1.21) [11], it can enter target cells very efficiently [12]. HI443, is a rationally designed, non-nucleoside, reverse-transcriptase inhibitors (NNRTIs) based microbicide (Figure 1B) with potent anti-HIV activity against the wild-type and drug-resistant HIV-1 isolates [13,14]. HI443 is a highly lipophilic molecule with a log P value of 4.39 [14]. It has nanomolar activity against primary HIV isolates, stable against a broad range of temperature and pH conditions [15] and in the vaginal environment, non-cytotoxic, non-inflammatory in the presence of human genital track epithelial cells, and has no adverse effect on human sperm motility [13,12]. Effectiveness and safety of STP [11,16], and HI443 [14,13] alone and in combination [17] as a potential anti-HIV vaginal microbicides has already been proven.

Some of the previous studies described the analysis of STP [16,18–20] and HI443 [14] using high performance liquid chromatography (HPLC) method. However, there is no information available about the analytical validation of the reported methods of STP and HI443. In addition, in our knowledge there is no analytical method for describing the quantitative analysis of STP and HI443 in nanoformulations intended for vaginal applications. Thus, there is a definite need to develop an efficient, sensitive and validated chromatographic method that can be used for simultaneous quantification of STP and HI443 in bulk or in their microbicide formulations.

**Figure 2:**
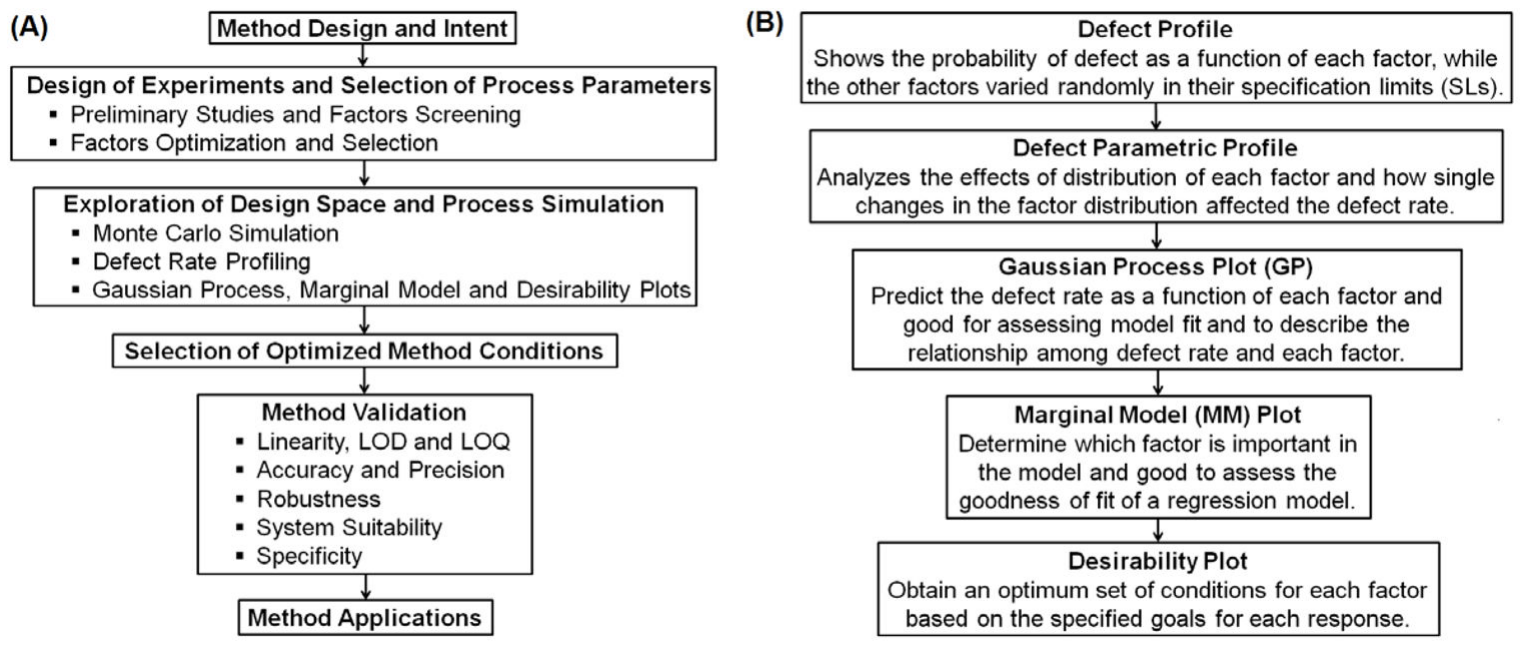
(A) Systematic experimental flow chart followed in this study. (B) Significance of the analyses generated in Monte Carlo simulation experiments.

By considering these aspects, a systematic approach was applied (Figure 2A) to develop a liquid chromatography (LC) method using experimental design and simulation approaches for the simultaneous analysis of STP and HI443 in bulk and in solid lipid nanoparticles (SLNs) formulated in this study. The Plackett-Burman (PB) and Box- Behnken (BB) experimental designs were used to screen, and evaluate the main and interaction effects of various factors on the selected responses. The Monte Carlo simulation was then applied using the Gaussian process (GP), marginal model (MM) and desirability plots to get the minimal DR of the process. The SLNs were characterized for their size distribution, and the drug encapsulation and release kinetics were analyzed using the developed HPLC assay.

## Materials and Methods

### Chemicals

The HPLC grade acetonitrile and methanol were purchased from Sigma Aldrich (St. Louis, MO, USA). The Milli Q water used in this study was obtained through Millipore water purification system (Millipore Corporation, Danvers, MA, USA). All other chemicals were of analytical grade and used as obtained from the suppliers.

### Instrument and chromatographic conditions

The HPLC system (Waters, Milford, MA, USA) consisted of a 1575 binary pump, 717 plus auto sampler, 2487 dual wavelength absorbance detector, and a Phenomenex Luna C_18_ column (150×4.6 mm, 3 μm). The results were acquired and processed with Breeze^™^ software (version 3.3). The assay was performed at ambient temperature (23°C) conditions. The mobile phase solvents were degassed using ultrasonic bath sonicator (Model 150 D, VWR International, West Chester, PA, USA) for 10 min before their use. The optimal composition of the mobile phase was based on the results obtained through experimental design and simulation methods.

### Preliminary studies and factor selection

Preliminary investigations were carried out to select initial HPLC analysis conditions. The analysis was performed using working standard solutions of STP and HI443 prepared by appropriate dilution of the stock solution of drugs (1 mg/mL) in acetonitrile with the initial HPLC mobile phase. The pK_a_ value of both drugs was determined using ACD I Lab software version 5.0.0.184 (ACD/Labs, Toronto, ON, Canada). The analysis was performed using a Phenomenex Luna C_18_ column due to its high stability in a broad pH range (pH 2–9) and in the presence of various organic solvents [21]. The feasibilities of various solvent systems involving acetonitrile, methanol, and water in different compositions of isocratic or gradient elution modes and flow rates were measured to obtain a good peak shape and resolution of both drugs. It has been observed that the gradient elution with acetonitrile provides better separation with good peak shape and shorter run time (preferred in this study, as it facilitates the analysis of several samples per day) [22], compared to the isocratic elution.

The preliminary observations revealed that the acetonitrile ratio during the gradient flows was critical for higher resolution and good peak shape of both drugs. The UV absorption maxima for STP is at 265 [18] and 268 nm [19], whereas for HI443 the absorption maxima is at 275 nm [14]. Therefore, it is important to identify a single wavelength to be used in this method at which both drugs have higher UV absorbance that means higher HPLC peak area. The sample mass overload, when the numbers of drug molecules are higher than the number of active sites in the stationary phase [23], could result in broader peaks with tail ends. Generally, the stationary phase of any HPLC column has a definite number of active sites that interact with the active drug molecules [23]. Thus, sample injection volume and concentration of drugs are important in method development. The mobile phase flow rate is also a critical parameter for low solvent consumption and some other advantages. After the preliminary investigations, acetonitrile ratio in the mobile phase, detection wavelength, mobile phase flow rate, and sample injection volume were selected as the independent variables (factors) with their low, medium, and high levels as showed in Table 1. The HPLC peak area and tailing (USP Tailing) of STP and HI443 were selected as the responses (dependent variables) in the screening design.

### Design of experiments

JMP^®^-Release 8.0.2.2 software (Cary, NC, USA) was used to perform the experiment design, simulation, DR profiling and data analysis.

#### Factors screening through Plackett-Burman (PB) design

The PB design [24,25] with 12 runs was initially used to estimate the main effects of five factors selected after preliminary investigations (Table 1). To provide a measure of process stability and inherent variability, three center points were also added in the PB design. The purpose of the PB design was to identify the significant effects of factors on the selected responses with the least number of runs as possible.

#### Factor optimization using Box-Behnken (BB) design

The BB design [24,25] with 12 runs and three center points was used in the optimization phase to evaluate the main and interaction effects of the significant factors selected after PB screening design to further optimize the HPLC analysis conditions.

#### The simulation and DR analysis

The Monte Carlo simulation was used to determine the distribution of process outputs as a function of random variation in the selected factors [25]. The Monte Carlo simulation was used since, it is the most efficient approach to determine the propagation of distributions in the process [26], as applied in analytical applications [27]. The simulation analyzes the DR of a process with respect to variation of the factors. The lower and higher specification limits (SLs) for each response is provided and carried over into the simulation output. The lower SL was selected for peak areas of STP and HI443; whereas, the higher SL value of 1.5 was applied for USP Tailing of both drugs according to the acceptance criteria for USP Tailing factor (<2) [28]. The simulation experiment was performed by specifying 15,000 runs to determine the DR of the process. The significance of all the analyses used in Monte Carlo simulation is explained in Figure 2B. The DR calculation was based on the number of runs specified in the simulation experiment and the SLs provided for each factor. This is given by Eq. (1):

(1) DR=m/n

Where, *m* is the number of defects produced at each point in the SL provided for each factor and n is the total number of runs in the Monte Carlo simulation [25].

### Method validation

The HPLC method was validated according to the ICH:Q2R1 guidelines [29]. The lowest concentration in the given range of linearity of both drugs was considered as the limit of quantification (LOQ); whereas, determination of limit of detection (LOD) values was based on the signal-to-noise (S/N) ratio of 3:1. The precision and accuracy of the method were assessed by using three quality control (QC) samples (low, medium, and high) covering the specified range of linearity of STP (0.195–25 μg/mL), and HI443 (0.098–12.50 μg/mL). The precision was reported in terms of percent relative standard deviation (%RSD); whereas, accuracy was reported in terms of percent mean recovery. The acceptance criteria for precision was that the %RSD <2% at each concentration level, while for accuracy the percent mean recovery should be in the range of 90–110%. The robustness was assessed with the following changes in the optimized method parameters: flow rate of the mobile phase (adjusted by ± 0.1 unit), initial gradient acetonitrile ratio (adjusted by ± 2 units), and detection wavelength (adjusted by ± 2 units). The variation in the HPLC peak area was calculated and the acceptance criterion of %RSD <2% was considered for each robustness parameter. The system suitability test was carried out by performing replicate injections of the standard solution (n=6) containing STP (10 μg/mL) and HI443 (5 μg/mL). The acceptance criteria were: %RSD for peak area and retention time <2%, resolution >2, USP Tailing <2, and number of theoretical plates >3000. The stability of the standard stock solution of drugs stored at 2–8°C for one month was evaluated by comparing with the freshly prepared solution of drugs at the same concentration. The stability evaluation was based on the calculation of HPLC peak area.

### Application of the developed HPLC method

#### Quantitative determination of STP and HI443 from solid lipid nanoparticles (SLNs)

Blank and drug loaded SLNs were prepared by using a phase-inversion method [30]. The SLNs were analyzed for their particle mean diameter (PMD; nm), and size distribution by dynamic light scattering (DLS) method using a Zetasizer Nano ZS (Malvern Instruments Ltd., Worcestershire, UK). To analyze the percent drug encapsulation efficiency (%EE), the SLNs were freeze-dried (Labconco Corp., Kansas City, MO, USA) and dissolved in acetone to degrade the SLNs. The acetone was evaporated at room temperature and the remaining sample was dissolved in the HPLC mobile phase and injected into the HPLC system. The peak area of the resulting HPLC chromatogram was calculated and the amount of drug encapsulated inside the SLNs was determined using a linear regression equation as calibration curve.

*In-vitro* drug release analysis was performed by dispersing the SLNs in water, which was then transferred to a dialysis bag (Spectra/Por Float-A-Lyzer G2, MWCO 3.5–5 kDa), purchased from Spectrum Laboratories Inc. (Rancho Dominguez, CA, USA). This was placed inside a dialysis tube containing 20 mL of the aqueous ethanol solution (50% v/v) used as a release medium. The whole system was then agitated in a thermostatic shaking water bath (BS-06, Lab Companion, Seoul, Korea) at 60 rpm and 37°C. Aliquots of 100 μL solutions were taken from the release medium at different time intervals of 0, 15, 30, 60, 150, and 300 min and analyzed for the amount of drug released. Simultaneously, the fresh release medium was added at the same time intervals to maintain the sink condition.

The release kinetics of STP and HI443 was analyzed using various *in-vitro* drug release kinetic models such as zero order, first order, Higuchi, and Korsmeyer-Peppas models [31].

#### Determination of molar extinction coefficient (ε) of STP and HI443

The developed HPLC method was applied to estimate the molar extinction coefficient (ε) value of STP and HI443. According to Lambert–Beer’s law [32], the absorbance (*Ab*) of a substance depends on its *ε* value, path length (*l*), and molar concentration (c). The Lambert- Beer’s law equation [32] was rearranged to determine the *ε* value by HPLC analysis [33,22].

The *ε* value was also determined by spectrophotometric analysis using Genesys 10 Bio UV-vis spectrophotometer (Thermo Electron Scientific Instruments LLC, Madison, WI, USA) and compared with the values obtained by HPLC analysis. The Lambert-Beer’s law was applied and a calibration curve of both drugs was constructed between *Ab* in absorbance units (AU) on the *y*-axis and *c* on the *x*-axis. A linear regression data analysis was performed through the experimental points by setting the intercept of the calibration curve to zero, and the ε value was obtained from the slope of these calibration curves in the HPLC and UV-Vis analyses.

### Data analysis

Statistical analysis was performed by using Students t-tests. The *p* value of <0.05 was considered statistically significant. Experiments were performed in triplicate (n=3) unless otherwise specified, and the average (mean) value of the responses with their standard deviation (SD) were calculated.

## Results and Discussion

### Factors screening through PB design

**Figure 3:**
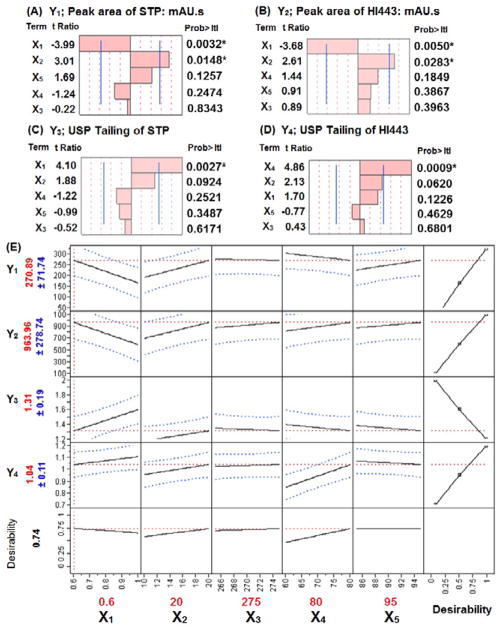
Pareto charts showing the standardized effect of factors, *X_1_* flow rate: mL/min, *X_2_* injection volume: μL, *X_3_* detection wavelength: nm, *X_4_* initial gradient acetonitrile ratio: % v/v, *X_5_* acetonitrile ratio at four minutes of gradient run: % v/v, in Plackett-Burman (PB) design for the responses: (A) *Y_1_* peak area of STP: mAU.s, (B) *Y_2_* peak area of HI443: mAU.s, (C) *Y_3_* USP Tailing of STP, (D) *Y_4_* USP Tailing of HI443. Bars extending past the vertical lines indicates the values reaching statistical significance (α=0.05). Asterisk (*) indicates the significant effect (*p*<0.05). (E) Prediction profiler and desirability plot in PB design.

The measured values for all the responses in PB design are summarized in supplementary material Table S1. The polynomial equations for the responses were developed after the data analyses in order to evaluate the best linear model for each response in relation to the factors (supplementary material equations, (1), (2), (3), and (4)). To understand the effect of the factors and their significance, Pareto charts were generated and showed in Figures 3A–3D, for the responses Y_1_, Y_2_, Y_3_, and Y_4_, respectively. Pareto chart analyses revealed that the factors X_1_ and X_2_ have significantly affected the responses Y_1_ and Y_2_. A negative relationship with X_1_ (flow rate; varied from 0.6 to 1.0 mL/min), and a positive relationship with X_2_ (injection volume; varied from 10 to 20 μL), was observed for the responses Y_1_ and Y_2_. The physicochemical meanings of the results observed here were consistent to the chromatographic principles and are explained below.

The HPLC peak area (*Ar*) is an integral of the absorbance (*Ab*) as a function of time. The *Ab* is concentration (*c*) dependent when a UV detector is used with the HPLC system. Concentration c is equal to the mass (*m*) divided by volume (*v*). If we consider that the amount (mass; *m*) of drug in each given injection into the HPLC system is constant, then *c* will be inversely proportional to *v*. But, for a given time *t, v* is multiplication of flow rate (*f*) and *t*. These parameters were all combined to give Eq. (2).

(2) $$Ar\;\alpha \frac{m}{f\times t}$$

It was noteworthy from Eq. (2), that if we decreased the value of *f*, *Ar* will increase for a given amount *m*. The *Ar* will also increase if we increase m at a constant value of *f* and *t*. A similar pattern was observed in this study as we increased *f* from 0.6 to 1.0 mL/min, *Ar* of STP and HI443 was decreased (Figures 3A and 3B). Also, when we increased the injection volume (that is *m* of the drug injected), *Ar* was increased as well.

The tailing of STP (Y_3_) and HI443 (Y_4_) was increased as X_2_ was increased from 10 to 20 μL. This could be due to the sample mass overload, which resulted in the tailing of the HPLC chromatogram [23]. The response Y_3_ was significantly affected by flow rate (X_1_) (positive correlation). Similar results for the correlation between the flow rate and peak tailing were observed in a previous study [1]. The response Y_4_ was significantly affected by X_4_ (positive correlation), which could be explained on the basis of partitioning of drug in stationary and mobile phases. Acetonitrile has a weaker interaction with the stationary phase silanol groups compared to water and methanol [34]. As the volume fraction of acetonitrile in the mobile phase was increased, more silanol groups were available for interaction with the drug molecule, which resulted in peak tailing. The tailing could be higher in the case of a more lipophilic and basic drug molecule as more silanol and stationary phase interactions would be observed [35]. Since HI443 is more lipophilic (log P: 4.39) [15] and has a higher acidic pka (~13) than that of STP (log P: 1.21; pKa~8.5) [11], the effect of acetonitrile was more significant for HI443 as explained above.

Based on the specified goals for each response the value of each factor was obtained as, X_1_=0.6 mL/min, X_2_=20 μL, X_3_=275 nm, X_4_=80% v/v and X_5_=95% v/v (Figure 3E) in the screening design. The factors X_3_ and X_5_ did not have significant effects on any of the responses, and based on the prediction profile plots (Figure 3E), 95% v/v for X_5_ and 270 nm for X_3_ was selected for optimization steps (BB design). Owing to their significant effects, factors X_1_, X_2_, and X_4_ in the PB screening design were considered as factors X_1_, X_2_, and X_3_, respectively, in the BB design to determine their interaction and quadratic effects on the responses Y_1_, Y_2_, Y_3_, and Y_4_.

### Factor optimization using BB design

**Figure 4:**
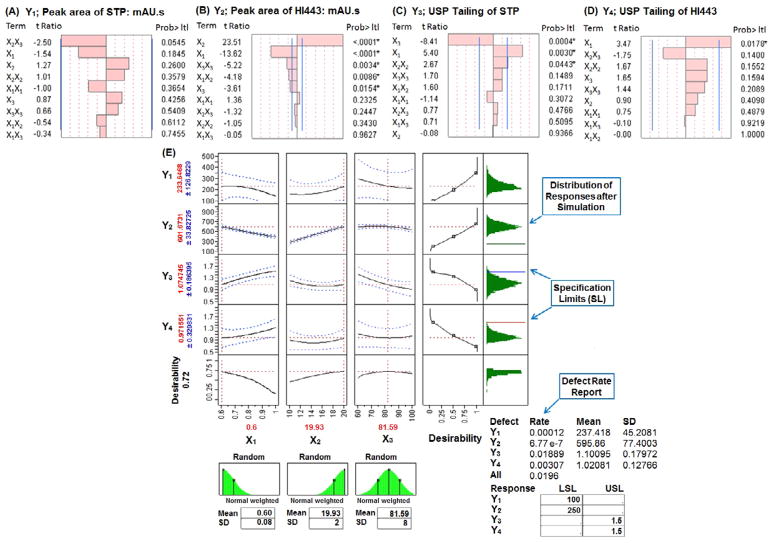
Pareto charts showing the standardized effect of factors, *X_1_* flow rate: mL/min, *X_2_* injection volume: μL, *X_3_* initial gradient acetonitrile ratio: % v/v, in Box- Behnken (BB) design for the responses: (A) *Y_1_* peak area of STP: mAU.s, (B) *Y_2_* peak area of HI443: mAU.s, (C) *Y_3_* USP Tailing of STP, (D) *Y_4_* USP Tailing of HI443. Bars extending past the vertical lines indicates the values reaching statistical significance (α=0.05). (E) Prediction profiler, desirability plot and simulation analysis showing the effect of factors, X_1_, X_2_, X_3_, and X_4_ in BB design for the responses, Y_1_, Y_2_, Y_3_, and Y_4_. Asterisk (*) indicates the significant effect (*p*<0.05).

The BB design with three levels, three factors, and 15 runs (Table 1) including three center points was used to optimize the HPLC analysis conditions. The measured values of the responses are summarized in supplementary material Table S2. Polynomial equations for each response are shown by supplementary material equations, (5), (6), (7), and (8)). To obtain an optimum set of conditions based on the specified goals for each response, desirability and prediction profile plots were generated based on the scale of desirability function as between d=0, for a completely undesirable, to d=1, for a fully desirable response [1]. Pareto charts (*t_critical_*=2.57) are shown by Figures 4A–4D, for the responses Y_1_, Y_2_, Y_3_, and Y_4_, respectively. Pareto chart analysis revealed that none of the factors had significantly affected the response Y_1_; whereas, Y_2_ has been significantly affected by several factors. The response Y_3_ has been significantly affected by factor X_1_ and X_2_; whereas, Y_4_ has been significantly affected by factor X_1_. The physicochemical meaning of these observations was based on the similar explanation as given for Eq. (2).

The relationship among factors and responses was further investigated by constructing a prediction profiler plot (Figure 4E) which gave the optimal values of; X_1_=0.6 mL/min, X_2_=19.93 μL (taken as 20 μL), and X_3_=81.59% v/v (taken as 82% v/v). To confirm these optimum set of conditions, six replicate (n=6) injections of standard solution containing STP (10 μg/mL) and HI443 (5 μg/mL) were analyzed to ensure if the observed values of the responses Y_1_, Y_2_, Y_3_, and Y_4_ were within the predicted range. It was observed that the difference between the observed and predicted value was <5%. Two randomly selected runs with X_1_=0.5 mL/min, X_2_=18 μL, X_3_=80% v/v and X_1_=0.7 mL/min, X_2_=16 μL, X_3_=85% v/v were also analyzed (n=3) in checkpoint analysis to ensure reproducibility of the model. The difference between the predicted and measured values was statistically insignificant (*p* value>0.05) and the percent error was <10%.

### Model simulation and DR profiling

**Figure 5:**
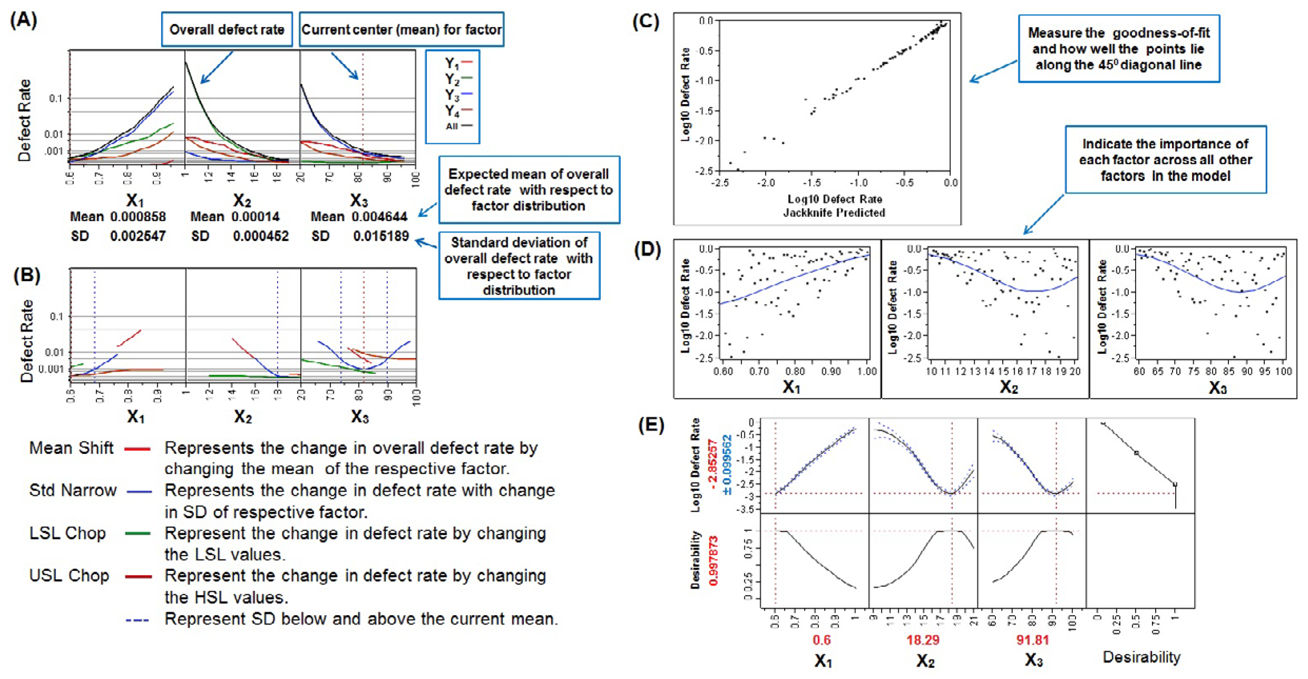
Defect rate analysis of the factors, *X_1_* flow rate: mL/min, *X_2_* injection volume: μL, *X_3_* initial gradient acetonitrile ratio: % v/v, for the responses, *Y_1_* peak area of STP: mAU.s, *Y_2_* peak area of HI443: mAU.s, *Y_3_*USP Tailing of STP, and *Y_4_* USP Tailing of HI443, shown by: (A) Defect rate profiler. (B) Defect parametric profiler. (C) Gaussian process plot. (D) Marginal model plots, (E) Prediction profiler plot for Log10 defect rate and desirability plot.

The overall DR of the process is calculated by using Eq. (1) was found to be 0.0196, indicating that about 1.96% of the HPLC analyses should be discarded (Figure 4E). This was very high for a process to be acceptable according to the Six Sigma guidelines for DR analysis [36]. The DR of 0.0196 was very close to DR value of response Y_3_ (0.0189), which indicate that most (about 96.43%) of the overall defects in the model were due to the Y_3_ variable. Defect profiler analysis (Figure 5A) showed the probability of defect as a function of each factor, while the other factors varied randomly in their SLs [25]. It was observed that the DR is directly proportional to X_1_ and inversely proportional to X_2_ and X_3_. This means that to reduce the DR, the value of X_1_ should be lowered while the values of X_2_ and X_3_ should be increased. The SD in these curves was related to the sensitivity of the DR with respect to the distribution of each factor. Since, SD of X_3_ was much higher than the SD of X_1_ and X_2_, improving the distribution in factor X_3_ would have the greatest effect in reducing the overall DR.

In order to visualize the effect of distribution of each factor, defect parametric profile curves were generated (Figure 5B). In these curves, the current DR for each response was represented in four ways corresponding to each of the four curves as explained in Figure 5B. To obtain the value of each factor that would have the minimal DR, a simulation experiment was performed with 15,000 runs using a Latin hypercube design with 80 numbers of design points and factor space of one [25]. The Latin hypercube design was first introduced by McKay et al. [37] and chosen here because, in this design, samples are evenly taken across the SLs provided and it gives different levels for a factor at each design point [37,38]. Overall 15,000 design points or runs were simulated and the proportion of points with responses out of the targeted SLs (corresponding to selected three factors X_1_, X_2_ and X_3_) was calculated. The Gaussian Process (GP) was then used to predict the DR as a function of each factor [25]. It produced three plots: the GP plot, marginal model (MM) plot, and desirability plot (as explained in Figure 2B, Figure 5C–5E, respectively). ‘In the GP plot, y axis contains the actual Log10 of DR values; whereas, x axis contains the corresponding predicted values (represented as Jackknife predicted values). The jackknife predicted values were determined using Jackknife method to obtain an unbiased prediction of goodness-of-fit [25]. The GP and MM plots showed the excellent model fit as points were very close to one another.

**Figure 6:**
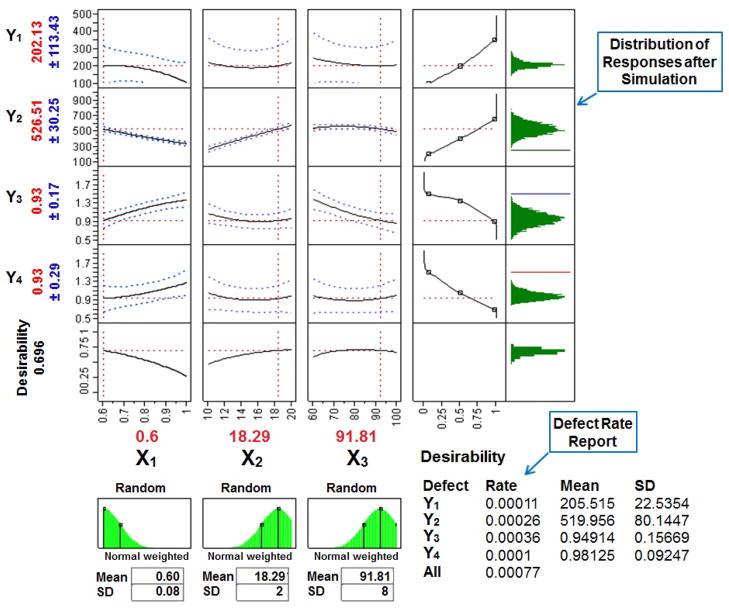
Prediction profiler, desirability plot and defect rate after Monte Carlo simulation, showing the effect of factors, *X_1_* flow rate: mL/min, *X_2_* injection volume: μL, *X_3_* initial gradient acetonitrile ratio: % v/v, for the responses, *Y_1_* peak area of STP: mAU.s, *Y_2_* peak area of HI443: mAU.s, *Y_3_* USP Tailing of STP, and *Y_4_* USP Tailing of HI443.

After the simulation experiments the optimal value of each factor producing a minimal DR of 0.077% (770 defects per million) was found to be: X_1_=0.60 mL/min, X_2_=18.29 μL, and X_3_=91.81% v/v (Figure 6). The overall DR was far improved and closer to the acceptable value of 3.4 defects per million as per Six Sigma guidelines [36] than the previous value of 19,600 defects per million (Figure 4E). Since, it was not appropriate to use the exact decimal values of the each factor in the HPLC analysis, the closest value of each factor, X_1_=0.6 mL/min, X_2_=18 μL, and X_3_=92% v/v, was selected for further analyses. The mobile phase gradient elution program selected after experimental design and simulation experiments was as follows (all solvent percentages were volume fractions): mobile phase A, water; mobile phase B, acetonitrile; time program, 0.01 min, 8% A/92% B; 4 min, 5% A/95% B; 7 min, 5% A/95% B; 8 min, 8% A/92% B; 10 min, 8% A/92% B.

### Validation of the developed HPLC method

**Figure 7:**
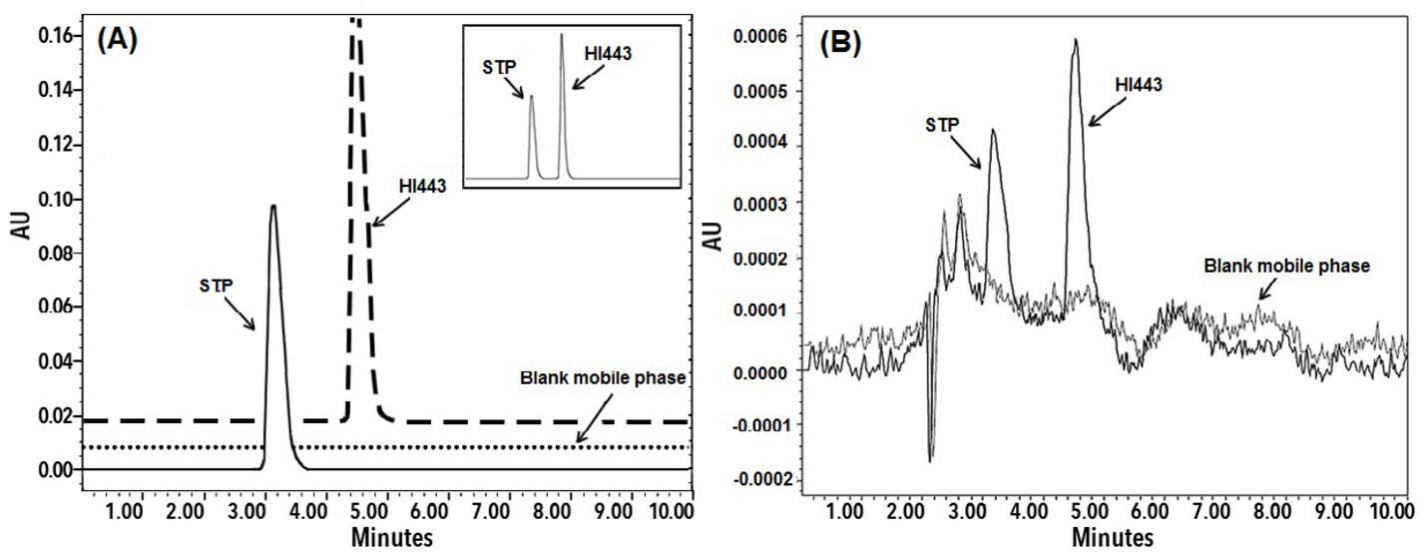
(A) Representative HPLC chromatogram of STP (10 μg/mL) and HI443 (5 μg/mL). The Figure in insert shows the HPLC chromatogram of the sample containing STP and HI443. (B) HPLC Chromatogram representing the peaks of STP (0.195 μg/mL) and HI443 (0.098 μg/mL) at their LOQ level.

The retention times of STP and HI443 using optimized HPLC analysis conditions were found to be 3.20 ± 0.15 and 4.60 ± 0.15 min, respectively, (n=6) as showed in Figure 7A. The calibration curves were found to be linear in the given concentration range of for STP and HI443, with coefficient of determination (*r*_2_) >0.99 for both drugs. The LOQ and LOD values were found to be 0.195 μg/mL and 0.098 μg/mL for STP, and 0.098 μg/mL and 0.049 μg/mL for HI443, respectively. Chromatogram representing the peaks of STP and HI443 at their LOQ level is shown in Figure 7B. The method was found to be accurate and precise (Table 2). The critical parameters determined for system suitability were well within the acceptance criteria (%RSD <2%). The method was found to be robust as the minor variations in the method parameters did not produce significant changes and was well within the acceptable limits (supplementary material Table S3). The drugs were found to be stable as no significant changes were observed in the peak area of the standard stock solution.

### Applicability of the developed HPLC method

#### Quantitative analysis of STP and HI443 in SLNs

**Figure 8:**
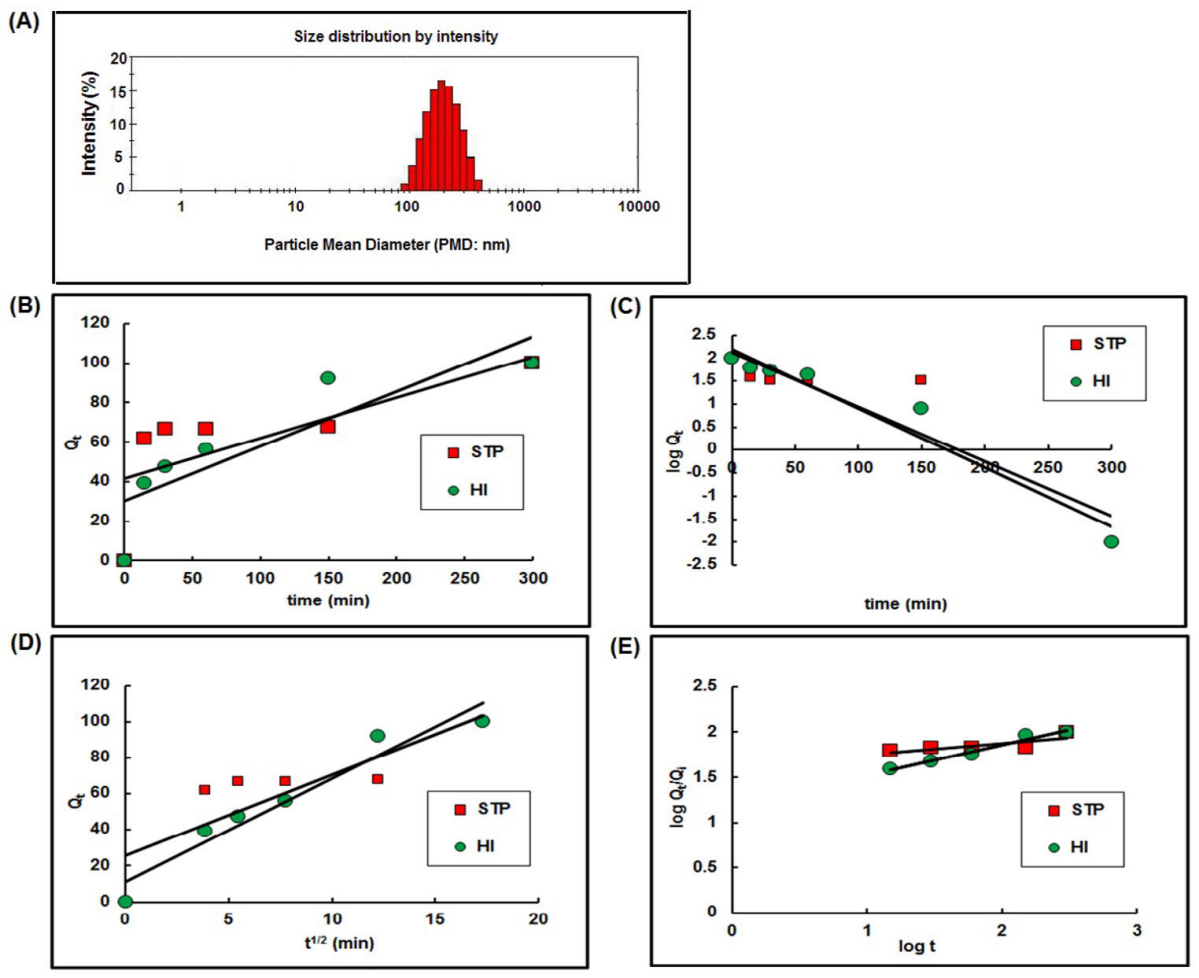
(A) Size distribution by intensity graph of solid lipid nanoparticles (SLNs). *In vitro* release kinetics of STP and HI443 from SLNs: (B) Zero order model, (C) First order model, (D) Higuchi model, (E) Korsmeyer-Peppas model.

The PMD of the SLNs was found to be 186.40 ± 7.83 nm (n=3) as showed by their size distribution profile in Figure 8A. The %EE of the SLNs was found to be 30.56 ± 9.44% w/w for STP and 94.80 ± 21.90% w/w for HI443 (n=3). The higher %EE of HI443 could be due to its higher lipophilicity than STP which favored it encapsulation inside the SLNs. The release kinetic curves are shown in Figures 8B–8E, for zero, first, Higuchi, and Peppas release models, respectively [32]. It has been observed that the STP release kinetics followed the first order model (concentration dependent), whereas HI443 followed the Peppas model (Table 3). The drug release mechanism from SLNs, the Korsmeyer-Peppas model was applied. It was observed that the drug release mechanism of STP and HI443 from SLNs followed Fickian diffusion since *n*<0.5 (Table 3) [31].

#### Molar extinction coefficient of STP and HI443

The calibration curves for both drugs using HPLC and UV analyses were found linear (r2>0.99). It has been observed that STP has lower *ε* value than HI443. The larger *ε* value of HI443 compared to STP corresponded to the higher *Ab* and *Ar* of HI443 as observed in UV and HPLC analyses, respectively, at the same concentration and at a given wavelength of 270 nm. As the HPLC method allowed for producing more accurate and reliable results and the drug peaks could be well separated from other potential impurities in the sample and selectively estimated compared to the UV analysis, the foregoing considerations finally governed the decision to use the ε values of 7,569.03 ± 217.96 L/mol/cm and 17,823.67 ± 88.12 L/mol/cm for STP and HI443, respectively, (n=3) at 270 nm. Overall, the concept explained here could be easily applied to determine the *ε* values of other bio-active molecules using HPLC method when there is a lack of availability of pure standards.

## Conclusions

A systemic experimental design approach has been applied to develop a simple and sensitive LC method for simultaneous quantification of topical microbicides, STP and HI443 in bulk and in SLNs. The PB and BB experimental designs were employed to effectively screen and optimize the main and interaction effects of selected factors. The Monte Carlo simulation was efficiently used to determine the optimal analysis conditions with a minimal defect rate. The combination of low flow rate with short run time minimized the amount of solvent consumption, and thus made this method highly economical. The method was found to have suitable applications in the determination of %EE, *in-vitro* release kinetics and ε values of both drugs estimated for the first time. The simulation method explained here can be effectively used to reduce the defect rate of a statistical process for quantitative analysis of functional molecules in bulk and pharmaceutical formulations using HPLC method.

## Supplementary Material

supplement

## Figures and Tables

**Table 1:** Variables with their corresponding values in the experimental design.

Variables	Levels		
	Low	Medium	High
Independent variables (factors)			
X_1_; Flow rate: mL/min	0.6	0.8	1.0
X_2_; Injection volume: μL	10	15	20
X_3_; Detection wavelength: nm	265	270	275
X_4_; Initial gradient acetonitrile ratio: % v/v	60	70	80
X_5_; Acetonitrile ratio at four minutes of gradient run: % v/v	85	90	95
Coded levels of levels of X_1_, X_2_, X_3_, X_4_ and X_5_	−1	0	+1
Dependent variables (responses)			
Y_1_; Peak area of STP: mAU.s			
Y_2_; Peak area of HI443: mAU.s			
Y_3_; USP Tailing of STP			
Y_4_; USP Tailing of HI443			

**Table 2:** Precision and accuracy of the method.

Drug	Conc. (μg/mL)	Precision		Accuracy	
		Intra-day (n=3)	Inter-day (n=3)	Intra-day (n=3)	Inter-day (n=3)
		%RSD^a^	%RSD^a^	Percent mean recovery ± SD^b^	Percent mean recovery ± SD^b^
STP	25	1.78	0.95	99.42 ± 1.77	94.68 ± 0.90
	6.25	0.66	1.34	107.80 ± 0.71	104.80 ± 1.40
	0.78	0.68	0.58	98.94 ± 0.67	90.27 ± 0.53
HI443	12.5	0.21	0.99	100.10 ± 0.21	98.72 ± 0.98
	6.25	0.55	1.00	99.86 ± 0.55	97.69 ± 1.07
	0.39	0.71	1.17	108.30 ± 0.77	101.60 ± 1.19

a Relative standard deviation

b Standard deviation

**Table 3:** Kinetics of in-vitro release profile of STP and HI443 from SLNs.

Drug	Zero order release model		First order release model		Higuchi release model		Korsmeyer-Peppas model		
	*K_0_*^a^	*r^2^*^b^	*K^1^*^a^	*r^2^*^b^	*K_H_*^a^	*r^2^*^b^	*k*^a^	*n*^c^	*r^2^*^b^
STP	0.21	0.52	0.027	0.83	6.63	0.46	0.21	0.11	0.65
HI443	0.28	0.75	0.030	0.94	6.68	0.90	0.07	0.26	0.97

a Rate constant: *K*_0_ in μg/min; *K*_1_ in min^−1^, *K_H_* in μg/min^1/2^, k in min^−1^.

b Square of the correlation coefficient.

c Exponent explaining the drug release mechanisms and classified as Fickian diffusion (n ≤ 0.5), case-II transport (*n*=1), anomalous transport (0.5<*n*<1), and super case-II transport (*n*>1) [31].

## Appendix A. Supplementary material

Supplementary material associated with this article can be found in the online version.
