# Supplementary material for: Application of Design of Experiment and Simulation Methods to Liquid Chromatography Analysis of Topical HIV Microbicides Stampidine and HI443
Source: J Anal Bioanal Tech. Author manuscript; Available in PMC 2015 Mar 10. (PMC4354943; doi:10.4172/2155-9872.1000180)
Supplement: supplement [file NIHMS604952-supplement.docx]

**Supplementary Material**

**Application of Design of Experiment and Simulation Methods in Liquid Chromatography Analysis of Vaginal Microbicides Stampidine and HI443**

Vivek Agrahari, Jianing Meng, Tao Zhang, Bi-Botti C. Youan^*^

*Laboratory of Future Nanomedicines and Theoretical Chronopharmaceutics, Division of Pharmaceutical Sciences, School of Pharmacy, University of Missouri-Kansas City, 2464 Charlotte Street, 690.71Kansas City, MO 64108, USA*

**^*^**Corresponding author. *E-mail address*: [youanb@umkc.edu](mailto:youanb@umkc.edu)

Tel.: +1 816-235-2410; Fax: +1 816-235-5779.

**Factors screening through Plackett-Burman (PB) experimental design**

The polynomial equations, (1), (2), (3), and (4), were developed after data analyses for the responses Y_1_, Y_2_, Y_3,_ and Y_4_, respectively, are as follows:

Y_1_ = 175.62 – 52.56X_1_ + 39.61X_2_ – 2.84X_3_ – 16.29X_4_ + 22.22X_5_ (1)

Y_2_ = 476.40 - 188.48X_1_ + 133.52X_2_ + 45.57X_3_+73.46X_4_ + 46.55X_5_ (2)

Y_3_ = 1.49 + 0.15X_1_+ 0.07X_2_ – 0.04X_3_ – 0.04X_4_ –0.022X_5_ (3)

Y_4_ = 0.94 + 0.03X_1_+ 0.04X_2_ + 0.01X_3_ + 010X_4_ – 0.02X_5_ (4)

The values on the *x*-axes of the Pareto charts represent the standardize effects, which are in fact the ratio of estimate and the standard error of the factor effect (*t* ratio values). The obtained *t* value is compared with a tabulated critical *t* value (*t_critical_* = 2.26, shown with the vertical line in the Pareto charts). This *t*_critical_ value was determined at *α* = 0.05 for residual degrees of freedom (*df* = number of runs *–* number of terms *–* 1). In Fig(s). S1a - 1d, the absolute *t* values of the factors whose length of the chart passed the vertical line (*t_critical_* at *p ˂* 0.05 and *df* = 9) have significant effects.

**Table S1:** Plackett-Burman (PB) design showing factors with their corresponding measured responses.

| Run | Factors | | | | | Measured responses ± SD (n = 3) | | | |
| --- | --- | --- | --- | --- | --- | --- | --- | --- | --- |
|  | **X_1_^a^** | **X_2_^b^** | **X_3_^c^** | **X_4_^d^** | **X_5_^e^** | **Y_1_^f^** | **Y_2_^g^** | **Y_3_^h^** | **Y_4_^i^** |
| F1 | +1 | +1 | +1 | -1 | -1 | 161.76±1.75 | 433.08±17.10 | 1.92±0.08 | 0.94±0.03 |
| F2 | +1 | -1 | -1 | -1 | +1 | 96.73±1.89 | 155.70±1.13 | 1.61±0.07 | 0.75±0.01 |
| F3 | -1 | +1 | -1 | -1 | +1 | 289.11±0.08 | 539.70±3.75 | 1.25±0.03 | 0.91±0.13 |
| F4 | -1 | -1 | +1 | -1 | -1 | 116.91±0.90 | 340.90±8.29 | 1.31±0.03 | 0.76±0.00 |
| F5 | 0 | 0 | 0 | 0 | 0 | 159.88±0.77 | 363.11±3.45 | 1.52±0.02 | 0.93±0.01 |
| F6 | 0 | 0 | 0 | 0 | 0 | 157.86±0.70 | 359.53±0.93 | 1.51±0.02 | 0.93±0.01 |
| F7 | +1 | +1 | -1 | -1 | -1 | 190.74±2.59 | 333.41±3.42 | 1.73±0.02 | 0.91±0.02 |
| F8 | +1 | +1 | +1 | +1 | +1 | 147.87±0.17 | 425.97±4.90 | 1.59±0.06 | 1.17±0.03 |
| F9 | -1 | +1 | +1 | +1 | -1 | 240.91±3.93 | 1008.03±13.95 | 1.27±0.12 | 1.05±0.03 |
| F10 | -1 | -1 | -1 | +1 | -1 | 141.86±0.79 | 393.27±5.26 | 1.32±0.02 | 0.97±0.02 |
| F11 | +1 | -1 | -1 | +1 | -1 | 91.32±0.83 | 243.62±2.28 | 1.56±0.01 | 1.14±0.01 |
| F12 | -1 | +1 | -1 | +1 | +1 | 284.10±10.25 | 1092.51±10.66 | 0.98±0.01 | 0.95±0.00 |
| F13 | -1 | -1 | +1 | -1 | +1 | 319.32±5.85 | 788.06±2.57 | 1.34±0.03 | 0.84±0.00 |
| F14 | +1 | -1 | +1 | +1 | +1 | 73.06±1.23 | 308.95±1.48 | 1.36±0.02 | 0.97±0.03 |
| F15 | 0 | 0 | 0 | 0 | 0 | 162.89±0.78 | 360.33±4.15 | 1.48±0.00 | 0.93±0.01 |

^a^ Flow rate: mL/min. ^b^ Injection volume: µL. ^c^ Detection wavelength: nm. ^d^ Initial gradient acetonitrile ratio: % v/v. ^e^ Acetonitrile ratio at four min of gradient run: % v/v. ^f^ Peak area of STP: mAU.s. ^g^ Peak area of HI443: mAU.s. ^h^ USP tailing of STP. ^i^ USP tailing of HI443.

**Factor optimization using Box-Behnken (BB) design**

The polynomial equations, (5), (6), (7), and (8) for the responses, Y_1,_ Y_2,_ Y_3,_ and Y_4,_ respectively, are as follow:

Y_1_ = 165.13 – 31.29X_1_ + 25.82X_2_ + 17.63X_3_ – 15.58X_1_X_2_ – 9.87X_1_X_3_ – 71.91X_2_X_3_ – 29.78X_1_X_1_ + 30.30X_2_X_2_ + 19.63X_3_X_3_ (5)

Y_2_ = 370.18 – 73.90X_1_ + 127.52X_2_ – 19.58X_3_ – 32.09X_1_X_2_ – 0.38X_1_X_3 –_ 10.11X_2_X_3_ + 10.84X_1_X_1_ – 8.36X_2_X_2_ – 41.69X_3_X_3_ (6)

Y_3_ = 1.26 + 0.16X_1_ – 0.25X_3_ + 0.07X_1_X_2_ + 0.03X_1_X_3_ + 0.03X_2_X_3_ – 0.05X_1_X_1_ + 0.12X_2_X_2_+0.08X_3_X_3_ (7)

Y_4_ = 0.93 + 0.18X_1_ + 0.05X_2_ + 0.09X_3_ – 0.01X_1_X_3_ – 0.13X_2_X_3_ + 0.06X_1_X_1_ + 0.13X_2_X_2_ + 0.11X_3_X_3_ (8)

In these equations, the positive and negative coefficients before the terms demonstrated an increasing and decreasing effect on the selected responses, respectively.

**Table S2:** Box-Behnken (BB) design showing factors with their corresponding measured responses.

| Run | Factors^a^ | | | Measured responses ± SD (n = 3) | | | |
| --- | --- | --- | --- | --- | --- | --- | --- |
|  | **X_1_^a^** | **X_2_^b^** | **X_3_^c^** | **Y_1_^d^** | **Y_2_^e^** | **Y_3_^f^** | **Y_4_^g^** |
| F1 | -1 | -1 | 0 | 122.87±7.04 | 274.35±20.24 | 1.16±.05 | 1.03±0.02 |
| F2 | -1 | 0 | -1 | 185.36±7.12 | 429.54±15.89 | 1.43±0.03 | 0.80±0.01 |
| F3 | -1 | 0 | +1 | 182.50±5.19 | 400.87±6.41 | 0.92±0.06 | 0.95±0.04 |
| F4 | -1 | +1 | 0 | 275.66±1.04 | 614.80±12.97 | 1.07±0.02 | 0.91±0.01 |
| F5 | 0 | -1 | -1 | 105.76±3.39 | 217.55±15.65 | 1.79±0.09 | 0.78±0.02 |
| F6 | 0 | -1 | +1 | 342.68±4.42 | 188.88±8.57 | 1.17±0.06 | 1.24±0.11 |
| F7 | 0 | 0 | 0 | 163.29±3.05 | 364.68±7.01 | 1.22±0.10 | 0.90±0.08 |
| F8 | 0 | 0 | 0 | 167.32±1.15 | 375.17±4.96 | 1.31±0.02 | 0.94±0.02 |
| F9 | 0 | 0 | 0 | 164.78±5.08 | 370.69±8.62 | 1.25±0.05 | 0.94±0.01 |
| F10 | 0 | +1 | -1 | 231.25±9.06 | 471.59±6.12 | 1.67±0.07 | 1.34±0.10 |
| F11 | 0 | +1 | +1 | 180.54±5.82 | 402.49±7.14 | 1.18±0.05 | 1.29±0.07 |
| F12 | +1 | -1 | 0 | 86.79±0.42 | 194.71±0.75 | 1.45±0.05 | 1.31±0.22 |
| F13 | +1 | 0 | -1 | 147.19±0.36 | 278.54±5.81 | 1.59±0.05 | 1.25±0.09 |
| F14 | +1 | 0 | +1 | 104.86±5.03 | 248.36±6.82 | 1.20±0.01 | 1.37±0.06 |
| F15 | +1 | +1 | 0 | 177.25±3.29 | 406.78±5.61 | 1.63±0.09 | 1.19±0.02 |

^a^ Flow rate: mL/min. ^b^ Injection volume: µL. ^c^ Initial gradient acetonitrile ratio: % v/v.

^d^ Peak area of STP: mAU.s. ^e^ Peak area of HI443: mAU.s.

^f^ USP tailing of STP. ^g^ USP tailing of HI443.
